# Non-classical protein secretion in bacteria

**DOI:** 10.1186/1471-2180-5-58

**Published:** 2005-10-07

**Authors:** Jannick D Bendtsen, Lars Kiemer, Anders Fausbøll, Søren Brunak

**Affiliations:** 1 Center for Biological Sequence Analysis, BioCentrum-DTU, Building 208, Technical University of Denmark, DK-2800 Lyngby, Denmark

## Abstract

**Background:**

We present an overview of bacterial non-classical secretion and a prediction method for identification of proteins following signal peptide independent secretion pathways. We have compiled a list of proteins found extracellularly despite the absence of a signal peptide. Some of these proteins also have known roles in the cytoplasm, which means they could be so-called "moon-lightning" proteins having more than one function.

**Results:**

A thorough literature search was conducted to compile a list of currently known bacterial non-classically secreted proteins. Pattern finding methods were applied to the sequences in order to identify putative signal sequences or motifs responsible for their secretion. We have found no signal or motif characteristic to any majority of the proteins in the compiled list of non-classically secreted proteins, and conclude that these proteins, indeed, seem to be secreted in a novel fashion. However, we also show that the apparently non-classically secreted proteins are still distinguished from cellular proteins by properties such as amino acid composition, secondary structure and disordered regions. Specifically, prediction of disorder reveals that bacterial secretory proteins are more structurally disordered than their cytoplasmic counterparts. Finally, artificial neural networks were used to construct protein feature based methods for identification of non-classically secreted proteins in both Gram-positive and Gram-negative bacteria.

**Conclusion:**

We present a publicly available prediction method capable of discriminating between this group of proteins and other proteins, thus allowing for the identification of novel non-classically secreted proteins. We suggest candidates for non-classically secreted proteins in *Escherichia coli *and *Bacillus subtilis*. The prediction method is available online.

## Background

The secretion of proteins across biological membranes is in most cases mediated by translocation machinery recognising a specific sequence tag or motif in the protein to be secreted. In bacteria, the classical tripartite structured Sec signal peptide governs most of the targeting to the secretion pathway. In addition to this Sec-dependent secretion, various other secretion pathways have been discovered, which work in a Sec-independent fashion. Most predominant is the twin-arginine translocation (Tat) secretion pathway where a twin-arginine consensus motif is located within the signal peptide itself [1,2]. While the Sec- and Tat-dependent secretion pathways translocate proteins across only the inner membrane in Gram-negative bacteria, additional translocation machinery components are found in the outer membrane of this group of organisms. The N-terminal signal peptide plays a central role in these secretory systems as the tag signalling secretion.

Surprisingly, some bacterial proteins have been found to be secreted without any apparent signal peptide. This phenomenon, termed non-classical secretion, was identified in eukaryotes approximately 15 years ago, when interleukin 1*β *and thioredoxin were found to be secreted despite being devoid of any identifiable signal peptide [3-5].

Some proteins, which have been found to display a function in the cytoplasm, have also been shown to actively participate in biological processes in the extracellular environment [6]. This does not imply that the function they uphold in the extracellular environment is identical to that in the cytoplasmic environment. Such proteins, which display two unrelated functions, have been named "moonlighting" proteins [7,8].

The detection of non-classically secreted proteins in the extracellular environment could obviously be attributed to cell lysis during experimental handling. However, some of the proteins have been detected extracellularly by different groups in several bacterial species supporting the argument that they are, indeed, exported from the intact cell.

Non-classically secreted proteins can be identified through inactivation of Sec-dependent secretion by mutation or chemical treatment. Hirose *et al*. used SecA mutants to disrupt the translocation machinery, thereby identifying several non-classically secreted proteins in *B. subtilis *[9]. Under such conditions, secretion must occur in a Sec independent manner.

It is currently unknown whether secretion by non-classical means occurs at a specifically localised membrane microdomain as seen for secretion of SpeB in *Streptococcus pyrogenes *[10]. Indeed, the mechanism or mechanisms responsible for non-classical secretion are unknown.

### Examples of non-classical secretion in bacteria

The first published study of non-classical secretion in bacteria reports the secretion of glutamine synthetase (GlnA) in the human pathogen *Mycobacterium tuberculosis *– one of the most important bacterial pathogens studied and responsible for millions of fatalities each year [11,12]. GlnA has been shown to be localised solely to the cytoplasm of the non-pathogen *Mycobacterium smegmatis *(although this difference need not be related to the pathogenicity of *M. tuberculosis*). A recombinant GlnA from *M. tuberculosis *expressed in *M. smegmatis *is also secreted, indicating that the signal for export is contained within the protein sequence [12].

For many years it has been known that *M. tuberculosis *secretes antigenic proteins without apparent signal peptides. ESAT-6 (early secretory antigenic target) is a small 6 kDa protein secreted by a novel secretion mechanism, the underlying details of which are still unknown. Another protein belonging to the same family, the small 10 kDa protein CFP-10, has subsequently been found to be secreted in spite of not possessing a signal peptide either (reviewed in [13]). The RD1 gene cluster in *M. tuberculosis *seems to encode the secretory system responsible for the secretion of the small antigenic proteins [14,15].

Unfortunately, the field has not yet agreed on a name for the new secretion system, although Stanley *et al*. designate the secretion system Snm for ***s***ecretio***n ***in ***m***ycobacteria [14]. Snm1 (Rv3870), Snm2 (Rv3871) and Snm4 (Rv3877) mutants are defective in secretion of ESAT-6 (EsxA) and CFP-10 (EsxB) and attenuated in virulence. The Snm1 and Snm2 proteins are part of a subfamily of ATPases containing an AAA domain, which is associated with chaperone-like functions. All three proteins may constitute parts of the translocation machinery of the Snm system [14]. ESAT-6, CFP-10 and homologous proteins are reported to share a WXG motif as identified by PSI-BLAST [16]. Whether the WXG motif alone is sufficient for secretion is unknown. In *Staphylococcus aureus*, the ESAT-6 homologs (EsxA and EsxB), have been found to be secreted in a similar fashion [17].

Superoxide dismutase (SodA) is a protein regularly found in the cytoplasm of *M. tuberculosis*. It has also been reported to be secreted in the same organism, but does not contain a signal peptide [18]. Again as with GlnA, SodA is not secreted in the non-pathogenic mycobacterium *M. smegmatis *[18,19]. In *M. tuberculosis*, a protein required for superoxide dismutase secretion was identified and named SecA2 [20]; deletion of the *SecA2 *gene abolishes virulence of *M. tuberculosis *in mice. This suggests that this new secretory pathway plays a role in the export of virulence factors in Gram-positive bacteria [20].

SecA2-dependent secretion has also been demonstrated in another Gram-positive pathogen, *Listeria monocytogenes *[21]. In this study, ten proteins without a classical Sec-signal peptide were found to be secreted, and the authors furthermore examined whether these proteins were translocated to the cell wall or secreted to the extracellular medium. In addition to these ten proteins, seven proteins with signal peptides were found to be secreted in a SecA2-dependent manner. The SecA2 pathway thus seems to be involved in both signal peptide dependent and non-classical secretion. It has been speculated that the SecA2 secretion pathway could be analogous to Type III secretion of virulence factors commonly found in pathogenic Gram-negative bacteria. All the non-classically secreted proteins detected in *L. monocytogenes *clearly have a cytoplasmic functional role whereas the precise extracellular functionality of the proteins remains unknown.

Another example is the staphylococcal nuclease from *M. smegmatis*. It contains a signal peptide and is secreted but secretion also occurs when the signal peptide is removed by mutation [22]. Experiments indicated that the release of the staphylococcal nuclease to the extracellular environment was not due to cell lysis. Recchi *et al*. were not able to characterise components of the secretion apparatus, but since no functional signal peptide was present, secretion must have taken place in a non-classical fashion [22].

ClyA, a pore-forming protein displaying a cytotoxic effect in mammalian cells, does not carry an N-terminal signal peptide but is nevertheless released from *E. coli *via vesicles that pinch off from the outer membrane. The secretion of ClyA is independent of the five known secretion pathways (Type I-V) in Gram-negative bacteria, thus bacterial "membrane blebbing" could be a novel form of secretion in bacteria [23,24].

One of the most comprehensively studied Gram-positive bacteria, *B. subtilis*, is also capable of secreting proteins via one or more non-classical pathways. Various studies have tried to identify the entire proteome of *B. subtilis *by use of 2D-electrophoresis, mass-spectrometry and prediction methods [9,25,26], including proteins localised to the extracellular environment. Due to differences in experimental setup and laboratory conditions, the various proteomic studies in *B. subtilis *do not agree on its extracellular proteome. Recently, a review on protein secretion in *B. subtilis *suggested that signal peptide independent protein secretion in bacteria is perhaps more common than previously thought [27]. This review lists 24 proteins found in the extracellular environment without having classical Sec signal peptides. This list of extracellular proteins was compiled from studies having different experimental setups as different cellular conditions were investigated [9,25,28].

Through a structure-function analysis of the Foldase protein (PrsA), Vitikainen *et al*. discovered a number of seemingly non-classically secreted proteins in *B. subtilis*, although this was not their initial aim [28]. As PrsA is an essential chaperone in *B. subtilis *involved in post-translational folding of exported proteins, mutations in *prsA *might lead to unpredictable alterations in protein secretion and overall stability of the cell. As mentioned by the authors themselves, the modifications cause significant cell lysis making it difficult to assess the degree of true non-classical secretion in this study.

Proteins involved in carbohydrate metabolism (Eno, PdhB, PdhD and CitH) were identified as being extracellular by Vitikainen *et al*. [28], although none of these proteins have a known extracellular function. Proteins involved in metabolism of amino acids, RocA and RocF, were initially found by Antelman *et al*. [25] to be non-classically secreted, but only RocF was later identified by Vitikainen *et al*. [28].

The motility and chemotaxis protein Hag was initially identified by Hirose *et al*. [9] but later also FlgK and FliD were found to be localised extracellularly [25]. Each of these three proteins is known to possess extracellular functions. The detoxification proteins KatA and SodA were initially identified extracellularly being secreted from a SecA *B. subtilis *deletion strain [9]. Later, also YceD was found extracellularly in stationary-phase *B. subtilis *[25]. In *Legionella pneumophila*, it has been shown that the ortholog of one of these detoxification proteins, KatA, is critical for stationary-phase redox reactions in the periplasm [29].

Although the extracellular function of KatA in *B. subtilis *is unknown, KatA could have a similar role in stationary-phase *B. subtilis*. The elongation factor Ef-G and the protein folding chaperone GroEL has been identified in the extracellular environment both by Antelmann *et al*. [25] and by Vitikainen *et al*. [28].

These two proteins were not found in the SecA deletion study by Hirose *et al*. and their potential extracellular function is unknown [9]. Although GroEL has a cytoplasmic function, the GroEL homolog HspB has been shown to be actively secreted in stationary-phase *Helicobacter pylori *[30]. The phage related proteins XepA, XkgG, XkdK, XkdM and XlyA were all identified by Antelmann *et al*. [25] while XdkG was identified in [9]. CwlC is a protein involved in the metabolism of the cell wall and was identified extracellularly [25].

*B. subtilis *provides a good example of why inconsistencies in the reports of the extracellular proteome of a specific organism makes it difficult to produce a list of validated non-classically secreted proteins. The discrepancies can be attributed to experimental conditions as the extracellular proteome of any organism will vary depending on the state of the cell and the nature of its environment. Cell lysis and protein degradation are other sources of errors that are difficult to entirely account for in all assays.

### Characterising non-classically secreted proteins

Little is known about the dynamical aspects of the non-classical secretion apparatus and whether the above mentioned cases are secreted through one or more different secretion systems. The only common theme to the phenomenon is the extracellular presence of these proteins despite the lack of a recognisable signal peptide or other conserved motifs.

Proteins entering the non-classical secretion pathway cannot be correctly identified using prediction methods such as PSORT [31] or SignalP [32]. Due to the lack of any apparent sequence motif in the N-terminal region of the sequence, it is doubtful whether secretion depends on this part of the protein in the way it does for other secretion pathways. Neither can non-classically secreted proteins be identified by their sequence homology to either known classically secreted proteins or to cytoplasmic proteins, as non-classically secreted proteins often seem to have a cytoplasmic function as well as an extracellular functional role.

Prediction methods for functional classification have been developed that allows for classification of proteins based on many sequence-derived features. For example, the method has been applied to predict protein functional categories [33] and to determine whether or not a protein is cell-cycle regulated [34]. Classification is based on a neural network evaluation of calculated and predicted protein sequence features such as post-translational modifications, secondary structure, isoelectric point and sequence length [33-36]. The idea behind such a classification scheme is that proteins can be described by their feature characteristics instead of by their sequence. Typically, some features are shared among proteins with similar function or subcellular localisation, despite the fact that they display no overall sequence similarity. Each protein sequence is assigned a feature-profile, and a neural network algorithm is trained to classify the proteins based on their feature-profiles. To avoid situations where data is unavailable, all features are either calculated or predicted directly from the protein sequence, but experimentally determined features can, in principle, be used as well.

We have previously applied this method with success to the problem of predicting non-classical secretion in mammals [36] and have now extended this to bacteria as described here. To better cover the diversity of bacteria, we have developed two different methods: one trained on and suited for proteins from Gram-positive bacteria and one for proteins from Gram-negative bacteria. Both methods are available from our website [37].

We show that, indeed, bacterial non-classically secreted proteins can be described in terms of sequence-derived features and use that property to propose additional secreted proteins in *E. coli *and *B. subtilis*.

## Results and discussion

We have performed an exhaustive literature search and compiled a list of apparently non-classically secreted proteins (Table 1). Due to the sensitivity of protein detection techniques, it obviously cannot be excluded that some of the proteins detected in the extracellular environment originate from cell lysis, as we discuss further below.

Only one of the publications make a distinction between cell surface localised proteins and proteins dispersed in the extracellular medium [21]. Thus, only in these cases are we able to distinguish between cell surface localised proteins and other extracellular proteins (Table 1).

### No simple sequence motifs in proteins undergoing non-classical secretion

No simple sequence motifs have been found that target all the known examples of non-classical secretion to the extracellular environment. Pallen *et al*. found a short WXG sequence motif in the ESAT-6 family of proteins by use of a PSI-BLAST approach [16]. However, we were not able to identify this as a common sequence motif in all proteins known to be localised extracellularly (data not shown). We searched for a common sequence motif in the 22 non-classically secreted proteins in *B. subtilis *using both a Gibbs-sampling approach [38] and the TEIRESIAS Pattern Discovery Algorithm [39], but found no conserved motifs.

As an alternative strategy, we attempted to identify the non-classically secreted proteins by means of their specific biological and chemical properties or characteristics. We have done this with success for mammalian proteins resulting in the prediction method SecretomeP [36]. The SecretomeP method for mammalian proteins is based on the fact that secreted proteins share certain features regardless of the mechanism by which they are secreted. A combination of such features can be used to distinguish them from non-secreted proteins.

We calculated or predicted approximately seventy different features for each sequence and tested each of them for discriminatory value. Subsequently, those contributing most strongly to the predictive performance were combined in a neural network approach as described previously [33,36]. Due to the relatively small number of known non-classically secreted proteins, we used as positive training examples Sec-dependent secreted proteins (with the signal peptide removed) and validated that this approach will identify non-classically secreted proteins correctly as it did for mammalian proteins [36].

### Features characterising non-classically secreted proteins

Many sequence-derived features will be characteristic to proteins undergoing secretion as well as to cytoplasmic proteins. We therefore searched for combinations of features with discriminatory value. Using an iterative scheme, in which features were tested individually and in combination, we eventually obtained a set of features together that had optimal discriminatory power. Two different prediction methods were trained; one for Gram-positive bacteria and one for Gram-negative bacteria.

When used independently, the predictive performance of the six protein features selected by the iterative scheme ranges from very poor (for example based on threonine contents, which on its own obtains a correlation coefficient below 0.4) to fairly good (for example based on the amino acid composition network yielding a correlation coefficient just above 0.7). However, combining the features in a single network increases the performance and the robustness of the method considerably. When the network is provided with information about the different protein features simultaneously, it is capable of correctly classifying a protein as either secreted or non-secreted approximately 9 out of 10 times regardless of the pathway of secretion (for qualitative performance and evaluation, see below).

The SecretomeP method is also capable of discriminating cytoplasmic proteins from classical secretory proteins (carrying classical Sec signal peptides). We tested the method on the dataset from SignalP 3.0, which is a widely used prediction method for identification of classically secreted proteins carrying signal peptides [32]. 87.6% of the Gram-negative positive data set (secreted proteins) for SignalP 3.0 received a SecretomeP score above 0.5. 96.4% of the negative data set (cytoplasmic proteins) received a SecretomeP score below 0.5. 89.5% of the Gram-positive SignalP positive data set received a SecretomeP score above 0.5 and 94.7% of the corresponding negative SignalP data set received a SecretomeP score below 0.5 using the Gram-positive prediction method. This indicates slightly better identification of cytoplasmic proteins over secreted proteins for both bacterial methods. While these results are far from those obtained using SignalP 3.0, they nevertheless demonstrate the power of a feature-based approach. The correlation coefficient for the Gram-positive and Gram-negative feature-based method were 0.85 and 0.87, respectively. As mentioned above, Tat substrates constitute yet another class of secretory proteins. In contrast to the Sec-dependent secretion pathway, the Tat-dependent secretion pathway is capable of translocating fully folded proteins [1]. Tat substrates have signal peptides with a tripartite structure much like Sec signal peptides, and a prediction method for Tat substrates was recently published [40]. We tested the SecretomeP method on Tat substrates, and conclude from the results (not shown) that the method is unsuitable for the prediction of this class of secretory proteins. The folded conformation of Tat substrates during translocation may constrain these proteins with regards to the chemical and structural properties that we evaluate and interpret in SecretomeP.

**Figure 1:**
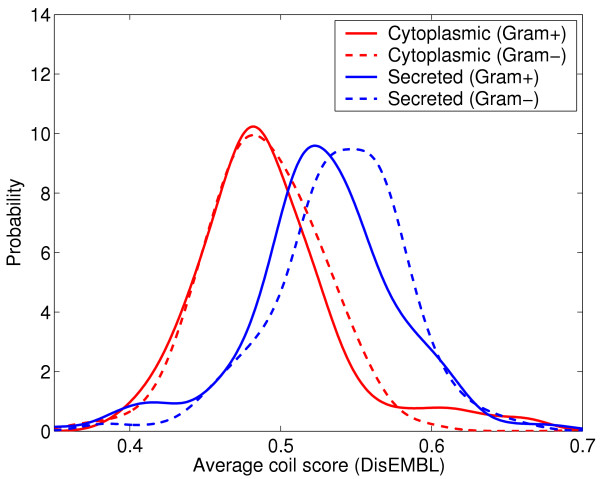
**Bacterial secreted proteins are more disordered in structure than cytoplasmic proteins**. The predicted number of coils (average per residue) by DisEMBL is higher for secreted proteins than for cytoplasmic ones. The tendency also holds for the two other measures of disorder predicted by DisEMBL (not shown).

An interesting observation regards the difference in output scores from DisEMBL [41] of secreted and cytoplasmic proteins. Secretory proteins show a greater degree of disorder for both Gram-negative and Gram-positive bacteria (Figure 1). Correspondingly, DisEMBL was chosen by the iterative scheme as an important feature for both prediction methods. This difference in disorder is an interesting observation, which could perhaps be attributed to a difference in the functional range of secreted proteins to that of cytoplasmic proteins.

Table 2a lists the features selected for the predictor for Gram-positive proteins. For prediction of Gram-negative non-classically secreted proteins, arginine contents, DisEMBL [41], instability index [42] and a specially designed amino acid composition network (see Materials & Methods) were selected as features (see Table 2b). Several amino acid composition based features were selected for both predictors confirming previous results, which have demonstrated the importance of amino acid composition in relation to this problem [31,32,43-46].

Others have published prediction methods for the subcellular localisation of proteins, but these were based solely on amino acid composition [44,47]. Furthermore, neither of the methods were developed with the aim of discovering non-classically secreted proteins, and neither of them seem to be publicly available (June 2005). Mammalian secretory proteins can also be classified to a certain extent based on amino acid composition [36].

Besides amino acid composition based features, structural features improve classification performance. Both PSIPRED (secondary structure prediction) and TMHMM (transmembrane helix prediction) were selected by the neural networks for their discriminatory value (see Methods section) when identifying secreted proteins from Gram-positive bacteria.

### Prediction results for known non-classically secreted proteins

Although the human pathogens *M. tuberculosis *and *M. smegmatis *are both from the phylum *Actinobacteria*, we have grouped them with the *firmicutes *as they stain positive in Gram staining. The tuberculosis causing *M. tuberculosis *has been studied intensively and a few proteins have been found to be secreted through a truly Sec independent pathway as described above.

The first case of non-classical secretion described in bacteria was a glutamine synthetase [12]. This protein [Swiss-Prot:POA590] was correctly predicted as secreted by our method. We tested a known cytoplasmic glutamine synthetase from *B. subtilis *[Swiss-Prot:P12425], which received a correct negative prediction with a score of 0.109. The localisation of glutamine synthetase to the cytoplasm in *B. subtilis *has previously been demonstrated [26].

Both the early secretory antigenic target proteins from *M. tuberculosis*, ESAT-6 and CFP-10, are classified as being secreted using the SecretomeP method. They obtain high scores of 0.557 and 0.813, respectively. The superoxide dismutase (SodA) is secreted via non-classical means in *M. tuberculosis*, whereas the homolog in *M. smegmatis *is not. However, a score indicating secretion of SodA from *M. tuberculosis *was not obtained.

Most of the reported examples of non-classical secretion in Gram-positive bacteria originate from *B. subtilis *(Table 1). The *B. subtilis *proteins with known extracellular functions (CwlC, FlgK, FliD, XepA, XkdG, XkdK, XkdM, XlyA) [27] receive high scores when evaluated by the prediction method except for two (Hag & XkdG).

The catalase KatA from *B. subtilis *was shown to be secreted independently of the Sec-secretion apparatus [9] under normal cellular conditions, but also during prolonged starvation [25]. KatA correctly receives a score of 0.759 in *B. subtilis *using the SecretomeP prediction method. In *Legionella pneumophila*, it has been shown that KatA is critical for stationary-phase redox reactions in the periplasm [29], correspondingly, *L. pneumophila *KatA scores 0.935.

The *E. coli *cytotoxin ClyA [Swiss-Prot:P77335] is the only reported example of non-classical protein secretion in *Proteobacteria*. The prevalence in other phyla prompted us to train a prediction method for Gram-negative bacteria as well. As for Gram-positive bacteria, we have determined a combination of discriminative features, which allows for correct classification of secretory and cytoplasmic proteins.

Despite being able to test on only one true example of non-classical secretion from this group of organisms, we expect performance values on the order of those obtained for the prediction method for Gram-positive bacteria. Since the performance on the independent examples from Gram-positive bacteria meets our expectations from the cross-validated test sets, we have no reason to believe this will not be the case for the Gram-negative prediction method. However, the sole example, cytotoxin ClyA, receives a somewhat low prediction score of 0.225.

A number of mammalian sequences are known to be non-classically secreted [48]. We searched the proteomes of *B. subtilis *and *E. coli*, but most of the mammalian proteins have no close bacterial homologs.

An exception is the human thioredoxin family protein [Swiss-Prot:P10599], for which both bacterial strains have reasonably close homologs with approximately 30% shared amino acid residues. However, none of the bacterial homologs are predicted to be secreted using the SecretomeP prediction method.

PSORTb version 2 [49] classified correctly as 'extracellular' five of the proteins with known extracellular function, while the remaining were classified either as 'cytoplasmic' or 'unknown'. However, PSORTb was not developed with the aim of identifying non-classically secreted proteins.

### Secretion, lysis, or leak?

At least one of studies that report known cytoplasmic proteins in the extracellular environment suggests lysis as result of experimental handling to be the cause [28]. Furthermore, it has been observed that *B. subtilis *is capable of causing lysis to surrounding cells in order to postpone sporulation [50].

Several of the *B. subtilis *proteins reported to be detected extracellularly are very abundant in the cytoplasm [26]. This observation leads to the speculation that very abundant cytoplasmic proteins leak to the extracellular milieu. These proteins all receive low scores from the SecretomeP 2.0 method (Table 1). Nonetheless, several proteins are repeatedly detected in the extracellular environment – even in different species. We believe that these proteins could be secreted proteins even though the secretion system is unknown and that system could, indeed, be several different systems.

### Cross species comparison

**Figure 2:**
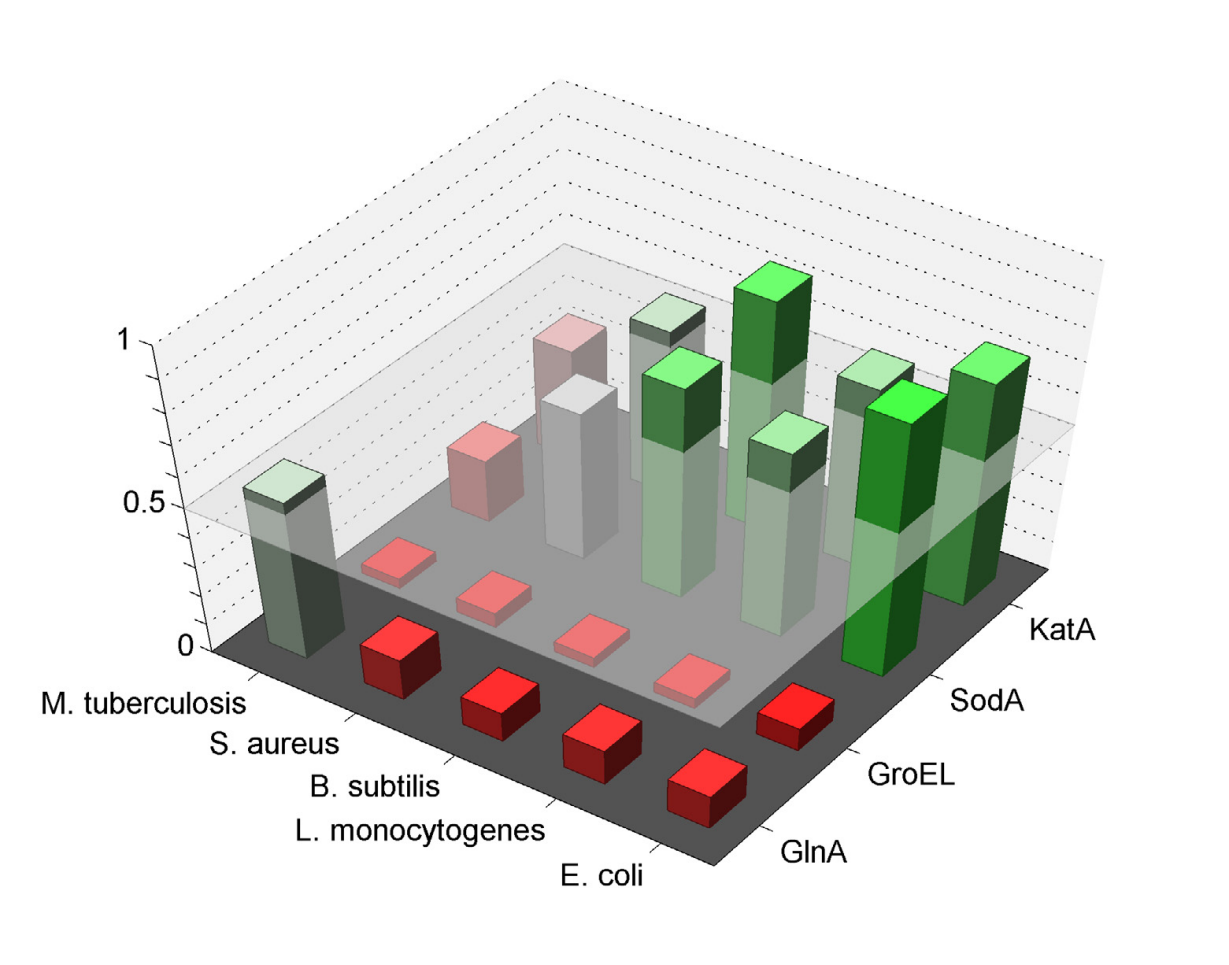
**Prediction on different proteins in different organisms**. Four proteins from five bacterial species. Scores above 0.5 indicate predicted secretion of that particular protein. For details, please refer to the text.

Prompted by the seemingly different localisation pattern of the superoxide dismutase (SodA) in different organisms, we have examined a few other proteins for the same property. Submitting homologous proteins from different bacterial species to the method revealed mostly similar secretion patterns. As seen from Figure 2, KatA is predicted to be secreted in four of the bacterial species investigated. To our knowledge, it is currently unknown whether KatA in *M. tuberculosis *has an extracellular function as observed for *L. pneumophila*.

The chaperone GroEL has been shown to have a "moonlighting" function in *L. monocytogenes *[21]. Whether this is an ubiquitous property of the protein is difficult to assess as all examined orthologs receive similarly low prediction scores. This could support the notion that GroEL is predominantly cytoplasmic. Finally, the glutamine synthetase (GlnA) which has a known extracellular role in *M. tuberculosis *[11,12], is predicted to have a cytoplasmic role in all the other bacterial species inspected here.

The Gram-negative pathogen *Pseudomonas aeruginosa *was recently submitted to a thorough computational and experimental analysis to determine its secretome. It was shown that 19.4% of the proteome is secreted [51]. Most of the secreted proteins were carrying a cleavable signal peptide. Using our method, a similar proportion (13.4%) of the *Pseudomonas aeruginosa *proteome obtains a score above 0.5 thus indicating secretion.

We inspected the proteomes of *E. coli *and *B. subtilis *for proteins entering a non-classical secretory pathway using SecretomeP. After having removed sequences with a predicted signal peptide using SignalP 3.0 [32], the 100 highest scoring proteins from both organisms were investigated. Lists from both bacterial strains contained many proteins with possible extracellular functions. For *E. coli*, we found that 51 of the proteins were hypothetical or with unknown function. Six were membrane-associated and eight were annotated as being related to flagellar function. The remaining 35 had other annotations.

For the Gram-positive bacterium *B. subtilis*, 56 of the 100 high scoring proteins had no functional annotation. Five sequences were annotated as membrane associated and four were involved in antibiotic resistance. The remaining 35 of the potentially non-classically secreted proteins in *B. subtilis *had other annotations. These lists are available as supplementary from our website [37].

Experimental verification of the predicted extracellular localisation of these proteins is obviously needed. As mentioned, non-classically secreted proteins have previously been observed to localise extracellularly only occasionally and have known cytoplasmic functions, thereby displaying functional roles in both environments. This means that designing an experiment, which matches the conditions required for the predicted proteins to migrate across the cellular membrane is not necessarily an easy task.

## Conclusion

We have compiled a list of bacterial proteins observed in the extracellular medium despite their apparent lack of a signal peptide. The list is based on an exhaustive literature search and we believe it to be almost complete. Non-classical secretion occurs in several different bacterial species and for a diverse group of proteins.

Furthermore, we present a novel method for prediction of non-classically secreted proteins in Gram-positive and Gram-negative bacteria. With high confidence based on a number of protein features, the method classifies as secreted most of the proteins reported to have an extracellular function. Coincidentally, most of the proteins which obtain low scores with our method have no currently known extracellular function. In summary, non-classical protein secretion is clearly supported by overwhelming evidence, and although the route(s) of export are still unclear, we are able to predict some of these proteins based on features which they share with classically secreted proteins. Other routes of export may allow a set of protein properties different from those of classically secreted proteins. This could explain the low scores some of the proteins reported to be non-classically secreted obtain with our method.

## Methods

### Generation of data sets

Ideally, our positive data set should consist of a large number of proteins secreted via non-classical pathways. Unfortunately, it was not possible to obtain a sufficiently large data set as only a small number of proteins undergoing non-classical secretion are known. Since we are looking for features shared among extracellular proteins, the mechanism by which a protein is secreted should not be important. We therefore used for training the large number of proteins known to be secreted via the classical Sec-dependent secretion mediated mechanism. All sequence data was extracted from Swiss-Prot release 44.0. Two individual training sets were created for *Firmicutes *and *Proteobacteria*, respectively.

A set of 690 extracellular proteins from *Firmicutes *(Gram-positive) and a set of 2185 extracellular proteins from *Proteobacteria *(Gram-negative) were extracted from the Swiss-Prot database based on annotations in the feature table (FT) and comments line (CC) [52]. Partial sequences were excluded from the data set. As we wanted to train a predictor that works in the absence of signal peptides, the signal peptide part of each sequence was removed according to the Swiss-Prot annotation. These lists of secreted proteins formed our positive data sets. Negative training sets were constructed by extracting 1084 proteins for *Firmicutes *and 2098 proteins for *Proteobacteria *from Swiss-Prot, which were annotated as localised to the cytoplasm. After redundancy reduction of the data sets based on a structural similarity criteria [53], 152 and 350 extracellular sequences were left in the positive data sets for *Firmicutes *and *Proteobacteria*, respectively. In the negative data sets, 140 and 334 sequences remained for *Firmicutes *and *Proteobacteria*, respectively. For Gram-positive bacteria (*Firmicutes *and *Actinobacteria*) a set of non-classically secreted proteins was retrieved from Swiss-Prot based on literature searches (see Table 1).

All data sets used are available as supplementary information from our website [37].

For identification of putative non-classically secreted proteins in *E. coli *and *B. subtilis*, we used the following accession numbers to extract the annotated and translated proteomes: [Genbank:NC_000913] for *E. coli *and [Genbank:NC_000964] for *B. subtilis*.

### Neural network architecture and feature integration

The construction of a non-classical secretion predictor based on protein features followed the scheme from [33,36]. Briefly, the procedure included: 1) Calculating and assigning the protein features for each protein sequence, 2) Encoding features for processing by a neural network, 3) Training neural networks using three-fold cross validation and various combinations of features, and 4) Determining the combination of features yielding the best performance based on correlation coefficient.

An extra feature predictor was constructed prior to network training. This feature was based on amino acid composition alone (inspired by Reinhardt and Hubbard [44]) and aimed at distilling into a single score information about the specific contents of all amino acids, thereby keeping the input dimensionality in feature space low. Care was taken to prevent hidden optimisation from taking place by ensuring that this feature followed the cross-validation scheme.

The prediction methods assigns a score to each protein between 0 and 1, where a score above 0.5 is considered indicative of secretion. 0.5 was chosen as the cut-off value for discrimination as this was the value used during training.

## Authors' contributions

JDB carried out sequence retrieval and drafted the manuscript. LK performed pattern finding, neural network training (with AF) and assisted with the drafting of the manuscript. AF was responsible for the amino acid composition network and the figure layout. SB provided general inputs and improvements to the manuscript.

## Figures and Tables

**Figure 3:**
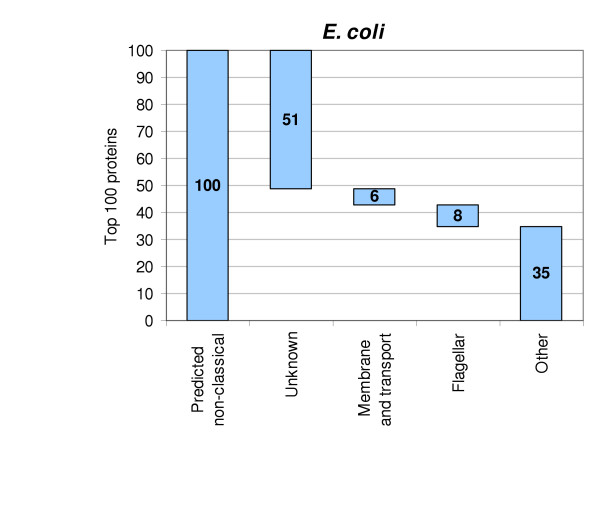
**Putative non-classical secretory proteins**. The top 100 scoring proteins are grouped based on annotation. Several groups have obvious relations to extracellular functions. For proteins with no annotation (grouped in 'Unknown'), our prediction method suggests an extracellular role. Proteins grouped in 'Other' may have an extracellular function, although this is not apparent in the current annotation.

**Table 1:** Non-classical secretory proteins in bacteria. The table lists proteins known to be localised extracellularly in Gram-positive bacteria. Many of the proteins are found localised to the cell surface, where as others are found in the surrounding media. Only one study distinguish between cell surface and extracellular localisation of the proteins [21]. ClyA is the only protein from Gram-negative bacteria reported to be non-classically secreted. Proteins listed above the horizontal line have known extracellular functions (see text for details). Abbreviations: Ex – Extracellular, Cs – Cell surface, BS – *B. subtilis*, MT – *M. tuberculosis*, LM – *L. monocytogenes*, EC – *E. coli*. ^*a*^Cytoplasmic abundance of proteins (% of total protein in the cell) in *B. subtilis *('-' no data available) [26].

**%^*a*^**	**Protein**	**Score**	**Function or similarity**	**Species**	**Location**	**Reference**
-	FliD	0.845	Flagellar hook-associated protein 2	BS	Ex	[25]
-	EsxB	0.813	CFP-10	MT	Ex	[13]
-	FlgK	0.795	Flagellar hook-associated protein 1	BS	Ex	[25]
-	XkdK	0.733	PBSX prophage gene	BS	Ex	[25]
-	XlyA	0.684	Amidase (PBSX prophage lysin)	BS	Ex, Cs	[25,54]
-	CwlC	0.634	N-Acetylmuramoyl-L-alanine amidase	BS	Ex, Cs	[25]
-	EsxA	0.557	ESAT-6, 6 kDa early secretory antigenic target	MT	Ex	[17]
-	XepA	0.545	PBSX prophage lytic exoenzyme	BS	Ex	[25]
-	XkdM	0.544	PBSX prophage gene	BS	Ex	[25]
-	GlnA	0.539	Glutamine synthetase 1	MT	Ex	[11,12]
-	ClyA	0.225	Cytotoxic protein	EC	Ex	[23,24]
1.27	Hag	0.218	Flagellin protein	BS	Cs	[9,25]
1.07	SodA	0.209	Superoxide dismutase [Fe] *M. tuberculosis*	BS, MT, LM	Ex	[6,9,18,28]
-	XkdG	0.090	PBSX prophage gene	BS	Ex	[9]

-	ManA	0.832	Phosphomannose isomerase	LM	Cs	[21]
-	KatA	0.759	Vegetative catalase 1	BS	Ex	[9,25,29]
-	SodA	0.701	Superoxide dismutase	BS	Ex	[9,28]
-	YceD	0.551	Similar to tellurium resistance protein	BS	Ex	[25]
-	DnaK	0.375	Heat shock protein	LM	Cs	[6,21]
-	PdhC	0.152	Pyruvate dehydrogenase (E2 subunit)	LM	Ex, Cs	[6,21]
0.71	PdhA	0.126	Pyruvate dehydrogenase (El *α *subunit)	BS	Ex	[28]
1.20	CitH	0.118	Malate dehydrogenase	BS	Ex	[25,28]
1.20	Gap	0.118	Glyceraldehyde-3-phosphate dehydrogenase	BS, LM	Ex, Cs	[6,9]
1.23	Eno	0.108	Enolase	BS, LM	Cs	[6,21,25,28]
-	RpoB	0.100	RNA polymerase *β *subunit	LM	Ex, Cs	[21]
-	RocF	0.082	Arginase	BS	Ex	[25,28]
5.17	EF-Tu	0.075	Elongation factor Tu	LM	Cs	[6,21]
-	RS9	0.073	Ribosomal protein S9	LM	Cs	[21]
-	RocA	0.070	Pyrroline-5 carboxylate dehydrogenase	BS	Ex, Cs	[25]
1.91	Ef-G	0.070	Elongation factor G	BS, LM	Ex, Cs	[6,25,28]
-	RpoC	0.059	RNA polymerase *β*' subunit	LM	Ex, Cs	[21]
0.76	PdhD	0.052	Pyruvate dehydrogenase (E3 subunit)	BS, LM	Ex	[6,25,28]
-	RL19	0.050	Ribosomal protein L19	LM	Cs	[21]
0.57	PdhB	0.047	Pyruvate dehydrogenase (El *β *subunit)	BS	Ex	[25,28]
1.30	GroEL	0.035	Class I heat shock protein (chaperonin)	BS	Ex, Cs	[21,28]

**Table 2:** Protein features found to be discriminative for identification of non-classically secreted Gram-positive (a) and Gram-negative (b) bacterial proteins

Feature	Program	Reference
Threonine contents		
Composition	see Materials & Methods	
Transmembrane helices	TMHMM 2.0	[55]
Gravy	ExPASy, ProtParam	[56]
Protein disorder	DisEMBL	[41]
Secondary structure	PSIPRED	[57]

Feature	Program	Reference

Arginine contents		
Composition	see Materials & Methods	
Instability index	ExPASy, ProtParam	
Protein disorder	DisEMBL	[41]
